# Clinical Practice Pattern of Immediate Intravesical Chemotherapy following Transurethral Resection of a Bladder Tumor in Korea: National Health Insurance Database Study

**DOI:** 10.1038/srep22716

**Published:** 2016-03-15

**Authors:** Gi Hyeon Seo, Jae Heon Kim, Ja Hyeon Ku

**Affiliations:** 1 Health Insurance Review and Assessment Service, Seoul, Korea; 2 Department of Urology, Soonchunhyang University Hospital, Soonchuhyang University Medical College, Seoul, Korea; 3 Department of Urology, Seoul National University Hospital, Seoul National University Medical College, Seoul, Korea

## Abstract

We evaluated the frequency and practice pattern of immediate postoperative intravesical chemotherapy (PIC) after transurethral resection of a bladder tumor (TURBT) in suspected non-muscle-invasive bladder cancer (NMIBC). Information from the Health Insurance Review and Assessment Service database from January 1, 2008 to December 31, 2013 was used. Patients with bladder cancer who received TURBT were considered as the cases (37,941 patients and 59,568 cases). The time of PIC after TURBT, types of PIC regimens, and the potential effect of PIC on the delay for additional treatment were analyzed. The study cohort included 23,726 subjects and 30,473 cases with a mean age of 66.8 ± 12.0 years, including 19,362 (81.6%) male patients. The rate of immediate PIC was 11.0% of cases (3,359 cases). There was significant difference in the frequency rate of additional treatment among patients with immediate PIC and patients without immediate PIC within 1 year from the first TURBT (15.2% vs 16.6%, p = 0.035). However, no difference was revealed for whole observational period (33.7% vs 34.5%, p = 0.373). The frequency rate of immediate PIC after TURBT for suspected NMIBC was low in real clinical practice. More efforts are needed to improve the usage rate of PIC after TURBT for suspected NMIBC.

Bladder cancer (BCa) is the 11th most frequent cancer worldwide, and in Korea, the number of BCa cases have increased from 2,180 cases in 1999 to 3,549 cases in 2011, a total of 37,950 cases during this period^1^.

Although the therapeutic impact of transurethral resection of a bladder tumor (TURBT) guarantees complete resection of a tumor in most cases of non-muscle-invasive bladder cancer (NMIBC), the recurrence rate of BCa is as high as 80%^2^. To date, various strategies have been suggested to prevent local recurrence of BCa following TURBT because treatments for recurrent BCa are associated with high costs. Moreover, BCa is the most expensive solid tumor to treat because of its recurrence especially for the NMIBC form^2^.

To reduce the recurrence rate after TURBT, single immediate postoperative intravesical chemotherapy (PIC) is recommended after each TURBT and exception is rare including suspected bladder injury or perforation^3^,^4^,^5^,^6^. PIC has been known to play a critical role in delay and prevention of tumor recurrence after TURBT by destroying circulating and residual tumor cells at the location of TURBT^7^.

Although there is some discrepancy about the degree of recommendation, both the American Urological Association (AUA) and the European Association of Urology (EAU) guidelines continue to recommend immediate PIC after TURBT^8^,^9^. Moreover, recent meta-analyses^10^,^11^ provide firm evidence for the effectiveness of immediate PIC by demonstrating lower likelihood of recurrence in suspected NMIBC. Although the current guidelines strongly recommend the use of immediate PIC after TURBT and meta-analyses support this recommendation, the real performance rate of immediate PIC is low. It has been recently demonstrated that there is a markedly low usage rate of immediate PIC after TURBT in NMIBC in both the United States (US) and European countries^12^,^13^,^14^,^15^.

A recent nationwide cross-sectional study in the US has shown a large disparity in the use of immediate PIC after TURBT among urologists^16^. The factors responsible for this large disparity include non-compliance of health providers and patients. Compliance of health providers is based on the surgeon’s decision or preference, surgeon’s volume, surgeon’s educational degree, nursing care, and preparation of pharmacy^15^,^16^.

Although Chamie *et al*.^12^ reported that Asian patients had a higher compliance rate than other races in terms of patient-related factors, no direct investigation has yet been performed to estimate the real under-usage rate of PIC in Asia including Korea.

We used a national data set to test this hypothesis. The National Health Insurance program in Korea was initiated in 1977 and it achieved universal coverage of the population by 1989. The Health Insurance Review and Assessment (HIRA) database covers the claims of 100% of the Korean population. Accordingly, the HIRA database contains information on almost all insurance claims, including prescribed medications and procedures, of the Korean population of approximately 50 million^17^. This is the first study to evaluate the real usage rate of immediate PIC after TURBT for suspected NMIBC using the national health database system and to investigate the potential role of immediate PIC in preventing or reducing the need for additional treatment of recurrence. Also, our ‘Discussion’ section focuses greatly on the fundamental reasons for the underusage of immediate PIC.

## Materials and Methods

### Data collection

Information from the Health Insurance Review and Assessment Service database from January 1, 2008 to December 31, 2013 was used. The HIRA database contains information on all insurance claims of 100% of the Korean population^17^. The HIRA service provided the data after patient de-identification. The data included age, sex, diagnosis, date of hospital visits, drug prescriptions received during inpatient and outpatient visits, hospital admissions, medical procedures, and emergency department visits. Drug information included brand name, generic name, prescription date, and duration and route of administration. This study was approved by the Institutional Review Board of Soonchunhyang University Hospital and Seoul National University Hospital.

### Study populations

**Figure 1:**
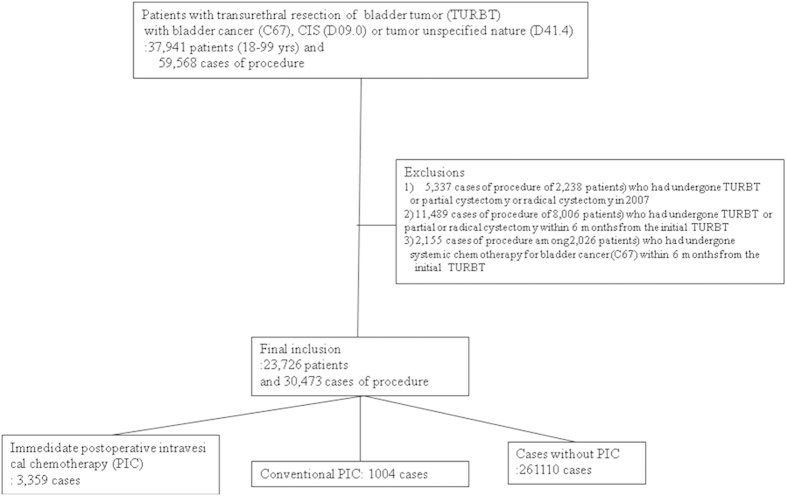
Subjects depositions.

Patient-level inclusion criteria included age (18–99 yrs). The study cohort that underwent TURBT included 37,941 patients and 59,568 cases of procedure. To include those patients with Ta bladder cancer and CIS, those patients with PIC regimens with CIS, bladder or bladder ca *in situ* (D09.0) and bladder tumor uncertain behavior or unspecified nature (D41.4) were added. Among the 37,941 patients, only 30,112 patients (49,454 cases of procedures) had a main claim for ICD 10 code of bladder cancer (C67), CIS (D09.0) or tumor unspecified nature (D41.4). The study cohort included ICD-10 code including bladder cancer (or CIS/tumor unspecified nature) among the patients who underwent TURBT. To define newly detected NMIBC, the patients (5,337 cases of procedure of 2,238 patients) who had undergone TURBT or partial cystectomy or radical cystectomy in 2007 were excluded. The patients (11,489 cases of procedure of 8,006 patients) who had undergone TURBT or partial or radical cystectomy within 6 months (180 days) from the initial TURBT were also excluded. The patients (2,155 cases of procedure among 2,026 patients) who had undergone systemic chemotherapy for bladder cancer (C67) within 6 months from the initial TURBT were also excluded. Finally, the study cohort included 30,473 cases of procedure among 23,726 patients (Fig. 1).

### Case definition

Procedure coding was used to investigate and define the study cohort including TURBT (R3512), partial cystectomy (R3470), radical cystectomy (R3481, R3482), intravesical instillation (R3655), and urethral or bladder irrigation (R3490). For intravesical chemotherapy regimens, mitomycin, epirubicin, doxorubicin, gemcitabine, and cisplatin were investigated. During exclusion of studying groups, we did not consider radiation treatment, because radiation treatment could not be performed alone in the treatment strategy of bladder cancer. The main exclusions were suspected advanced bladder cancer and recurred bladder cancer. The patients (11,489 cases of procedures among 8,006 patients) who had undergone TURBT or partial or radical cystectomy within 6 months from the initial TURBT were also excluded. The patients (2,155 cases of procedure of 2,026 patients) who had undergone systemic chemotherapy for bladder cancer (C67) within 6 months from the initial TURBT were also excluded. Those patients with systemic chemotherapy were defined by treatment with any of chemotherapy regimens for bladder cancer including gemcitabine, cistplatin, methotrexate, vinblastine, and doxorubicin. For gemcitabine, in cases of concurrent procedure codes with bladder irrigation or bladder instillation were regarded as PIC therapy.

### Study design

A retrospective cohort study design was used to estimate the real usage rate of immediate PIC. For suspected NMIBC cohort, the PIC rate was estimated and the timing of PIC was also evaluated. The type of PIC regimen was also investigated. To investigate the potential role of immediate PIC in preventing recurrence, disease-free intervals were estimated. Same investigation was also performed for post-operative BCG.

Index date was the day of admission for TURBT. Immediate PIC was defined by the cases that received PIC within admission and within 7 days. Considering the claiming lately and no conventional within 7 days from index date, including those patients with PIC within 7 days from index date is reasonable. Due to limited information from HIRA, the operation date could not be detected clearly within admission. Disease-free period was defined as the duration from TURBT until new TURBT or partial and radical cystectomy or systemic chemotherapy.

### Outcomes

Primary outcome was the frequency of immediate PIC use within 7 days from the index date. For secondary outcomes, the usage rates of PIC within 8 to 30 days, and 31 to 60 days (conventional PIC) from hospitalization were evaluated. Post-operative BCG usage rate was estimated. Moreover, the potential role of PIC or immediate PIC in preventing recurrence of bladder cancer was investigated by using estimation of disease-free period.

### Statistical analysis

Frequency analysis to estimate the frequency rate of PIC or BCG treatment was performed by R package software. A Poisson distribution was assumed to calculate the 95% confidence intervals (CIs) for odds ratio (OR) for disease-free period between patients with PIC or immediate PIC and patients without PIC.

## Results

The mean age of the study cohort was 66.8 + 12.0 years (median age = 68 years), including 19,362 male patients (81.6%). Among the total of 30,473 cases of TURBT procedure, single TURBT per patient was performed in 18,892 patients (79.6%). Double TURBT per patient was performed in 3,475 patients, triple TURBT was performed in953 patients, quadruple TURBT was performed in 291 patients, quintuple TURBT was performed in 87 patients, sextuple TURBT was performed in 24 patients, septuple TURBT was performed in 3 patients, and octuple TURBT was performed in 1 patients.

The frequency rate of additional operation or systemic chemotherapy after the initial TURBT within one year was 15.4% (3,442 cases). The frequency rate of additional operation or systemic chemotherapy after the initial TURBT within the whole observation period (until December, 2013) was 31.2% (6,989 cases).

The rate of immediate PIC was 11.0% of cases (3,359 cases). A total of 10.9% of cases (3,327cases) were treated within admission, and a total of 0.1% of cases (32 cases) were treated within 7 days at the outpatient department. Among the regimens of immediate PIC, mitomycin C was the most popular regimen (2,518 cases 75.0%). The next most commonly used regimens were epirubicin (834 cases, 24.8%), doxorubicin (5 cases, 0.1%), gemcitabine (2 cases, 0.1%), and cisplatin (0 cases, 0.0%). The frequency rate of PIC use within 60 days from the index date was 14.3% (4,363 cases). A total of 2.8% of cases (857 cases) were treated within 8~30 days from the index date, and a total of 0.5% of cases (147 cases) were treated within 31~60 days from the index date. Among the regimens of PIC within 60 days, mitomycin C, epirubicin, doxorubicin, gemcitabine, and cisplatin were 3,560 cases (74.3%), 1,152 cases (24.0%), 38 cases (0.8%), 38 cases (0.8%), and 6 cases (0.1%), respctively. Among the top five hospitals where TURBT is most commonly performed, the rates of immediate PIC were 3.9%, 5.6%, 5.7%, 22.6%, and 23.3%, respectively.

There was significant difference in the frequency rate of additional operation or adjuvant systemic chemotherapy among patients with immediate PIC and patients without immediate PIC within 1 year from the first TURBT (15.2% vs 16.6%, p = 0.035). There was no difference throughout the observation period (33.7% vs 34.5%, p = 0.373). With or without PIC within 60 days, there were no difference within 1 year (15.8% vs 16.6%, p = 0.230) or thoughout the observation period (35.6% vs 34.2%, p = 0.077).

For the frequency pattern of BCG instillation therapy, a total of 11.864 cases (38.9%) were treated with adjuvant BCG instillation therapy within 90 days from the index date. There was a significant difference in the frequency rate of additional operation or systemic chemotherapy according to adjuvant BCG instillation both within 1 year (15.0% vs 17.4%, p < 0.001) and throughout the observational period (31.7% vs 36.1%, p < 0.001).

## Discussion

Currently, there are two important issues related to the use of PIC in suspected NMIBC; the underusage rate itself and disparity among the urologists. This study shows the real usage rate of immediate PIC in Korea, and moreover, this study discusses the fundamental reasons for the low usage rate of immediate PIC and disparity in performing PIC.

Although both the AUA and EAU guidelines support the use of immediate PIC and recent meta-analyses also support the use of immediate PIC, the real performance rate of immediate PIC is still low. Although the recent report by Palou-Redorta *et al*. showed a relatively high usage rate of immediate PIC in European countries of 43.3%^15^, most studies have shown a low usage rate of immediate PIC following TURBT ranging from 0.32~3.2%. This study also shows a low usage rate of immediate PIC after TURBT in Korea. This is the first study to investigate the real usage rate of immediate PIC in Asian countries and it shows that there is a similar low usage rate and similar disparity in the PIC use pattern to that in Western countries.

Besides this low usage rate of immediate PIC, there also exists a large disparity in the clinical use pattern of immediate PIC. Recently, Cookson *et al*.^16^ showed nationwide heterogeneity in the clinical practice pattern of immediate PIC, and moreover, they reported that the majority of urologists in the US have no experience of immediate PIC. Only 2% of the urologists always use immediate PIC (100%), 17% of them use immediate PIC only on half of the occasions (50%), and the majority of urologists (67%) never use immediate PIC. Neither the geographical region nor the practice setting pattern (academic training hospital or private local hospital) was associated with immediate PIC usage patterns. This implies that individual disparities among urologists in terms of performing immediate PIC are responsible for the low usage rate of immediate PIC. In our data, among the top five hospitals where TURBT is most commonly performed, the practice pattern of immediate PIC showed a marked disparity (from 3.9% to 23.3%).

Immediate PIC following TURBT for all localized bladder cancers was strongly recommended by the EAU guidelines in 2011 and 2013 for low and intermediate risk group of bladder cancers and as an option for high risk group of bladder cancers^3^,^18^,^19^. The AUA guidelines support the use of immediate PIC after TURBT because it may decrease the risk of recurrence in NMIBC^20^. The possible mechanism for early recurrence of NMIBC following TURBT is implantation of floating bladder cancer cells into the healthy bladder epithelium, and immediate PIC has a protective effect against early implantation of cancer cells^7^.

There are many reasons for the low usage rate of immediate PIC in real clinical practice. Suggested reasons for underuse of immediate PIC include increased cost of extra post-operative nursing care after TURBT and difficulties in performing PIC in the operating room, pharmacy, and recovery room^16^.

However, there are more fundamental reasons for the low usage rate of immediate PIC. First of all, these EAU or AUA recommended guidelines could be considered impractical and not applicable because immediate PIC has to be performed before pathologic confirmation of cancer type and even confirmation of local staging. Many urologists are reluctant to use immediate PIC because they believe that the effectiveness of immediate PIC in intermediate or high risk tumors cannot be guaranteed^15^. Mischaracterization of the tumor stage may result in underestimation of recurrence risk in NMIBC. For instance, the final pathological stage is not consistent with the cystoscopic clinical stage in approximately 30% of cases^5^. This ambiguity at the time of surgery may influence the urologist’s decision regarding immediate PIC.

Another reason is that many urologists do not agree and do not perform the guideline recommendations. Chamie *et al*.^12^ reported that there was no significant change in the percentage of subjects who received compliant care after publication of clinical practice guidelines in their univariate analysis. This finding was further highlighted in provider-level compliance. Recently Burks *et al*.^21^ reported potential issues associated with the implementation of clinical guidelines, and they suggested that only disseminating the published guidelines is not adequate to formulate a treatment strategy and reduce the disparity but it is necessary to gain an understanding of the logistical and practical barriers to following the guidelines.

Moreover, although these two guidelines support the use of immediate PIC to prevent recurrence of bladder cancer after TURBT, the degree of recommendation is not consistent. The AUA guidelines justify the use of immediate PIC as an optional treatment only, weighing not so great important treatment strategy as EAU guidelines recommend. The AUA guidelines do not actually recommend the use of immediate PIC in all low risk groups of bladder cancer, and they state that the health outcomes of the interventions are not sufficiently well known to permit meaningful decisions, or preferences are unknown or equivocal. The AUA guideline also considers the negative issues of immediate PIC including the cost issues, non-confirmation of pathology, and the side effects of PIC regimens^22^,^23^. This inconsistency in the guidelines themselves also causes clinical disparity in use of immediate PIC.

Another reason is the studies that report about the negative role of immediate PIC. Although the recently updated systematic reviews or meta-analyses by Abern *et al*.^10^ and Perlis *et al*.^11^ support the use of immediate PIC after TURBT in terms of prolongation of recurrence-free intervals and reduction in the risk of early recurrence, there have been several studies with criticism about the effect of immediate PIC in suspected NMIBC^9^,^22^,^23^. The most important issue for the indication of immediate PIC is that it lacks level I positive evidence for multiple and recurrent tumors, high grade tumors, and disease progression.

Gudjonsson *et al*.^22^ reported in their prospective, randomized, multicentric study that use of immediate PIC after TURBT for NMIBC may reduce the likelihood of tumor recurrence. However, the positive benefits of immediate PIC were minimized in patients of the intermediate or high risk group. Moreover, Bohle *et al*.^24^ reported in a randomized, double-blind, placebo-controlled study that immediate PIC with gemcitabine after TURBT was not superior to placebo in terms of recurrence-free intervals. They concluded that improved TURBT techniques and continuous irrigation system have a more positive effect than immediate PIC in terms of recurrence-free survival^24^. Both studies suggested that the clinical benefit of immediate PIC in high-risk groups was minimal, and multiple instillation therapy could be indicated^8^,^22^. Similarly, Dobruch *et al*.^25^ concluded in their meta-analysis that immediate PIC may not be recommended routinely because the clinical benefit was small in terms of recurrence rates in patients with multiple tumors. This created great controversies among urologists with respect to guidelines. With respect to this issue, Abern *et al*.^10^ in their meta-regression analysis reported that individual tumor risk factors such as recurrence, multifocal pattern, high grade, and T1 stage, have no effect on the favorable clinical outcome of immediate PIC in NMIBC.

There are not only negative points in oncologic clinical issues but there also exist negative points in economic issues. Rao *et al*.^23^ reported that routine immediate PIC significantly lowered the overall cost for recurrence during hospitalization, but these benefits were diminished by contrary for when it has been performed in outpatient settings.

To overcome the low usage rate of immediate PIC and disparity in the clinical practice pattern, several tasks need to be performed. Current tasks for clinical urologists to treat suspected NMIBC are clear. There is an urgent need to encourage the use of immediate PIC for suspected NMIBC and to perform well-designed, randomized clinical trials (RCTs). Education could be a desirable solution to overcome the preconception or misunderstanding among urologists about immediate PIC with respect to the clinical benefit or side effects, One study revealed successful improvement in the rate of immediate PIC use by adopting reasonable educational protocols^21^. Burks *et al*.^21^ emphasized the importance of right implications to achieve quality improvement in care of NMIBC patients. They suggested a task such as a multidimensional approach that included clinician’s education, local logistic activities, and support for adequacy of pharmacy and staffing resources.

Perils *et al*.^11^ critically assessed the studies included in their recent meta-analysis using the important concept of risk-of-bias and quality-of-evidence assessment. Contemporary methodology suggests low quality of evidence for the examined outcomes. Therefore, RCTs with careful randomization and blinding are still warranted to clarify the real usefulness of immediate PIC in the NMIBC cohort.

Several limitations exist in our study. First of all, due to the limitation of data, the clear clinical stage could not be described in this study. Moreover, the exact date of TURBT could not be obtained; hence, immediate TURBT was defined when the patient was treated with TURBT during the same hospitalization period. It means that the critical concept of immediate PIC could not be adopted in this study; hence, the real usage rate of immediate PIC in our cohort could be even lower. Limited information was available regarding the surgical techniques, and patient comorbidities. A more critical point is that this study could not clearly discriminate between repeat TURBT and new TURBT for a recurrent tumor.

To date, immediate PIC following TURBT has a beneficial effect on the recurence with minimal adverse events. However, due to the low quality of evidence, well-designed RCTs with proper blinding and placebo are warranted. Besides, there is a lack of consensus on the real indications, the optimal chemotherapy agent, and optimal timing of PIC among urologists.

## Conclusion

Immediate PIC use after TURBT for suspected NMIBC was low in real clinical practice in Korea like in other countries. More efforts are needed to improve the usage rate of immediate PIC after TURBT for suspected NMIBC. Moreover, high-quality RCTs are warranted in the near future.

## Additional Information

**How to cite this article**: Seo, G. H. *et al*. Clinical Practice Pattern of Immediate Intravesical Chemotherapy following Transurethral Resection of a Bladder Tumor in Korea: National Health Insurance Database Study. *Sci. Rep.* **6**, 22716; doi: 10.1038/srep22716 (2016).
